# The Effects of Mucolytic Agents N-Acetylcysteine and Erdosteine on Hemostasis in Humans

**DOI:** 10.17691/stm2024.16.4.06

**Published:** 2024-08-30

**Authors:** S. Oktar, S. Doğru, S. Motor, M. Demirköse, E.Ş. Erden

**Affiliations:** MD, PhD, Associate Professor in Pharmacology, Department of Pharmacology; University of Health Sciences, Beyhekim Training and Research Hospital, Konya, Turkey; MD, Assistant Professor in Chest Diseases, Department of Chest Diseases; Gaziantep University, Medical School, Gaziantep, Turkey; MD, PhD, Associate Professor in Biochemistry, Department of Biochemistry; Mustafa Kemal University, Medical School, Hatay, Turkey; MD, Department of Chest Diseases; Ankara Atatürk Sanatory Education and Research Hospital, Ankara, Turkey; MD, Associate Professor in Chest Diseases, Department of Chest Diseases; Mustafa Kemal University, Medical School, Hatay, Turkey

**Keywords:** coagulation, platelet count, thrombocyte, N-acetylcysteine, erdosteine

## Abstract

There are some indications in a few studies on animals and humans that the mucolytic agents N-acetylcysteine and erdosteine have a disruptive effect on the coagulation cascade.

This study **aimed** to examine the effects of both erdosteine and N-acetylcysteine on the coagulation pathway.

**Materials and Methods:**

The research was conducted on patients treated with N-acetylcysteine (600 mg/day) or erdosteine (600 mg/day) for 7-14 days. To evaluate the coagulation and hemogram parameters, blood samples were taken from patients before treatment and on days 7-14 after starting the treatment. Hemogram and coagulation parameters were measured in the venous blood samples.

**Results:**

The levels of factor II significantly decreased, and the platelet count significantly increased in patients treated with erdosteine. D-dimer levels reduced and factor VII levels increased in patients treated with N-acetylcysteine, but these changes were not significant (p=0.069 and p=0.062, respectively). Other coagulation and hemogram values did not change in both groups. Erdosteine may have a dualistic effect on the coagulation cascade through its different metabolites.

## Introduction

N-acetylcysteine (NAC) and erdosteine belong to a class of medicines called mucolytics. These mucolytic agents have been widely used for many years in the treatment of patients with various respiratory problems [1]. These drugs showed clinical efficacy in acute respiratory diseases and in chronic obstructive pulmonary disease. Both agents are mostly used to treat chronic obstructive bronchitis and acute exacerbation of chronic bronchitis in adult and pediatric clinics dealing with chest diseases. A few studies on animals and humans report their disruptive effect on the coagulation cascade. In other studies, the researchers have shown on volunteers and patients that NAC has anticoagulant and platelet-inactivating effects [2-5]. There are studies on animals that have also demonstrated that NAC disrupts platelet aggregation [6]. Erdosteine in some animal studies was reported to produce a dualistic effect on hemostasis [7]. For example, a low dose of erdosteine (3 mg/kg) prolonged prothrombin time (PT) and international normalized ratio (INR) in young rats, and also clotting occurred in tubes. Additionally, there was a decrease in coagulation factor levels in animals treated with different doses of erdosteine [8-9]. On the other hand, we were unable to find any human study in the literature investigating the effects of erdosteine on the coagulation cascade. Therefore, we aimed to investigate the effects of erdosteine on hemostasis and coagulation parameters in humans. Mucolytic agents are generally used for a few weeks in pediatric patients and adults for mucolytic and especially expectorant purposes. Duration of treatment with the drug was not adequately considered in human studies with NAC. In this study, the effects of both NAC and erdosteine on hemostasis and coagulation were investigated, taking into account their commonly used dosage and treatment duration.

## Materials and Methods

### Subjects

This study was approved by the Mustafa Kemal University Local Ethics Committee (Hatay, Turkey) and the participants signed the written informed consent. The study was carried out in the Hospital of Mustafa Kemal University in Hatay. A healthy control group was not planned in this research protocol, so patients diagnosed with viral respiratory tract infection were included in the study. Patients who apply to the clinic are generally infected for 1–3 days. The patients were infected at the time the first blood sample was taken. Although there is some information that viral infections have a procoagulant effect, it is not clear [10]. In particular, the procoagulant effect of respiratory viral infections is on a lung tissue, and a systemic coagulopathy has not been proven [11]. Subjects who had a disease that affects coagulation, i.e., were using medications that influence coagulation, pregnant, or breastfeeding, were excluded from the study. A total of 30 patients were included in the study: 15 patients were administered erdosteine (600 mg/day) and 15 patients NAC (600 mg/day). Mucolytic treatment started during the active stage of the disease, and towards the end of the treatment, the active disease has regressed. For this reason, if there is a viral procoagulant effect, independent of the drug, it is expected to decrease [12]. A total of 19 patients (11 patients receiving erdosteine and 8 patients taking NAC) have completed the study. Both drugs were prescribed to the patients for 2 weeks. Blood samples were taken from the patients before the treatment. The 19 patients taking erdosteine and NAC were called for a follow-up examination within 7–14 days before they stopped taking the medicine. During the examination, venous blood samples were taken from these patients. Hemogram and coagulation parameters were measured in the blood samples.

### Biochemical analysis

The blood samples were put into a tube containing 3.8% sodium citrate (4.5 ml blood sample and 0.5 ml citrate mixture) for coagulation analysis. Part of the blood samples were put into a hemogram tube, and plasma samples were processed by a centrifuge at 3000 g for 10 min. Hemogram parameters such as hemoglobin, hematocrit, white blood cell count, and platelet count were measured using the BC-6800 auto hematology analyzer (Mindray, China). Hemostatic parameters such as INR, PT, and activated partial thromboplastin time (APTT) were measured by the MDA-II Automated Coagulation Analyzer System (BioMérieux, Inc., USA). Coagulation factors such as the D-dimer test, fibrinogen, factor II, factor VII, factor X, and antithrombin III (AT-III) were evaluated by the AMAX-200 Automated Analyzer (Trinity Biotech, Germany).

### Statistical analysis

Statistical analyses were performed using the Statistical Package for Social Sciences 24.0 for Windows (SPSS Inc., Chicago, IL, USA). The descriptive tests were used for determination of the coagulation and hemogram parameters in blood samples and demographic data. Although the number of subjects was small, the data distribution was generally normal according to the Shapiro–Wilk test. The paired t-test was used to compare normally distributed parameters. The Wilcoxon test was preferred to compare parameters that did not show normal distribution (INR, D-dimer, fibrinogen, and white blood cell count in the NAC-treated group and D-dimer, APTT, and AT-III in the erdosteine-treated group). Results are presented as mean ± standard deviation; p<0.05 was considered statistically significant.

## Results and Discussion

**Figure 1.**
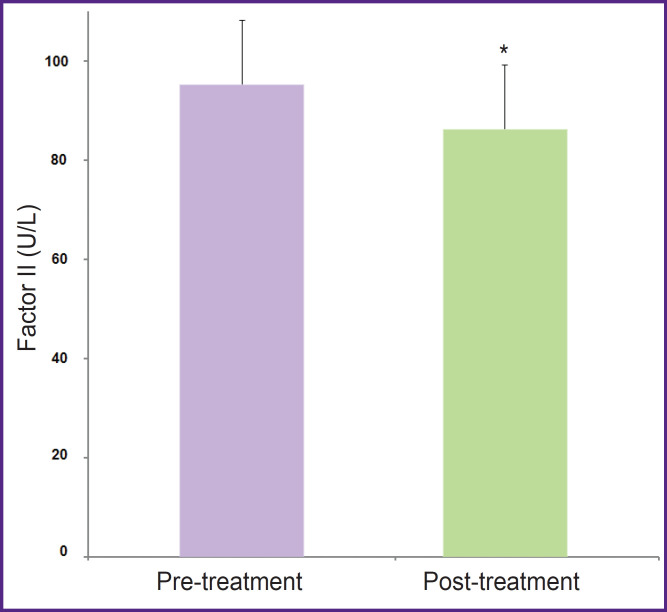
The levels of coagulation factor II in male patients (n=9) treated with erdosteine for 7–10 days (mean age — 34±18 years) Values are represented as mean ± standard deviation; *p=0.026

**Figure 2.**
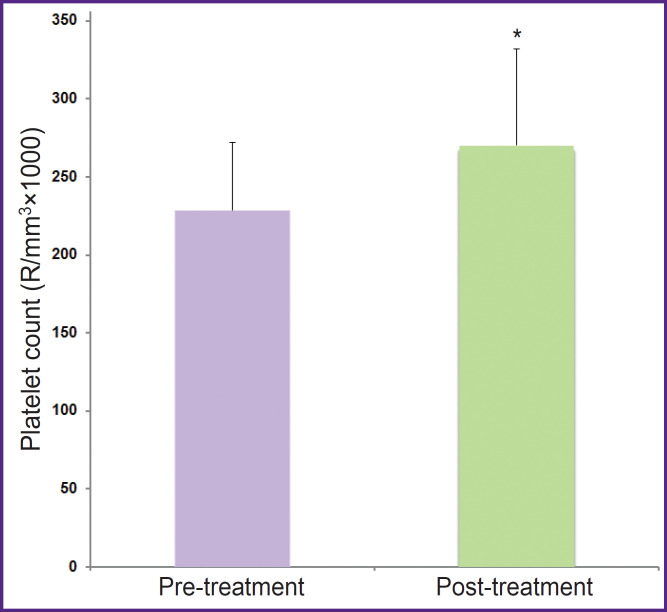
The platelet counts in male patients (n=9) treated with erdosteine for 7–10 days (mean age — 34±18 years) Values are represented as mean ± standard deviation; *p=0.007

The effects of NAC on the hemogram, hemostasis, and coagulation parameters are represented in Table 1. D-dimer levels reduced and factors VII levels increased, but these changes were not significant (p=0.069 and p=0.062, respectively). The other coagulation and hemogram parameters did not change in patients treated with NAC 600 mg/day for 2 weeks. Table 2 shows the hemogram, hemostasis, and coagulation parameters in the blood of subjects treated with erdosteine 600 mg/day for 2 weeks. The platelet count increased significantly, and factor II levels lowered insignificantly. The decrease in factor II levels was significantly pronounced in male patients treated with erdosteine (Figure 1), while the platelet count remained significantly high (Figure 2). The other parameters did not show any significant changes depending on gender.

**T a b l e 1:** The values of hemostatis and hemogram parameters in the patients (5 males and 3 females) treated with N-acetylcysteine for 7–14 days

Parameters	Pre-treatment	Post-treatment	p
INR (%)	1.06±0.11	1.00±0.07	0.235
D-dimer (μg/L)	1388±1634	690±544	0.069
PT (sn)	13.62±0.96	13.06±0.54	0.174
APTT (sn)	28.18±3.57	28.77±2.49	0.627
Fibrinogen (mg/dl)	446±193	428±90	0.889
Factor II (U/L)	100.50±15.09	97.63±16.41	0.581
Factor VII (U/L)	100.00±21.44	119.13±25.66	0.062
Factor X (U/L)	105.88±17.91	113.00±17.54	0.126
AT-III (pg/ml)	99.25±18.84	98.63±9.88	0.915
WBC count (K/mm^3^)	13.64±6.89	11.87±2.34	0.866
HGB (mg/dl)	13.49±1.29	13.59±1.39	0.337
HTC (%)	41.55±4.07	42.19±3.96	0.431
PLT count (K/mm^3^)	305.87±129.05	335.28±185.86	0.866

N o t e. Values are represented as mean ± standard deviation. INR: international normalized ratio; PT: prothrombin time; APTT: activated partial thromboplastin time; AT-III: antithrombin III; WBC: white blood cell; HGB: hemoglobin; HTC: hematocrit; PLT: platelet.

**T a b l e 2:** The values of hemostatis and hemogram parameters in the patients (9 males and 2 females) treated with erdosteine for 7–10 days

Parameters	Pre-treatment	Post-treatment	p
INR (%)	0.98±0.05	0.99±0.05	0.298
D-dimer (μg/L)	420±484	456±530	0.213
PT (sn)	12.61±0.69	12.75±0.69	0.242
APTT (sn)	29.48±3.69	30.74±5.21	0.266
Fibrinogen (mg/dl)	339±68	320±99	0.326
Factor II (U/L)	93.22±14.62	86.89±11.93	0.117
Factor VII (U/L)	93.45±21.13	91.85±19.57	0.805
Factor X (U/L)	100.91±20.29	95.91±16.30	0.254
AT-III (pg/ml)	106.36±26.41	105.09±31.43	0.838
WBC count (K/mm^3^)	9.71±3.27	8.48±2.88	0.201
HGB (mg/dl)	15.06±0.80	14.78±0.97	0.349
HTC (%)	44.83±2.40	43.99±2.26	0.274
PLT count (K/mm^3^)	233.40±44.61	274.11±58.84	0.004

N o t e. Values are represented as mean ± standard deviation. INR: international normalized ratio; PT: prothrombin time; APTT: activated partial thromboplastin time; AT-III: antithrombin III; WBC: white blood cell; HGB: hemoglobin; HTC: hematocrit; PLT: platelet.

The main findings of this study are that erdosteine treatment significantly reduces coagulation factor II, especially in men, and significantly increases the platelet count regardless of gender. Unlike erdosteine, NAC does not change the coagulation factors and the platelet count. On the other hand, in patiens treated with NAC, D-dimer partially decreased (1388±1634 vs 690±544; p=0.069) and there was a partial increase in factor VII level (100.00±21.44 vs 119.13±25.66; p=0.062).

Viral infections increase the risk of thromboembolism in humans regardless of the pathogen type. However, it has not been proven that this increase is not due to immomoblization [10]. Infuenza virus and other respiratory viruses are inflammatory and procoagulant *in vitro* [13]. Although influenza virus increases the tendency to thrombosis, there is no such clinical finding for respiratory syncytial virus and adenoviruses [14]. The virus-mediated procoagulant state returns to normal after two weeks [10, 15]. Experimental rhinovirus infection in humans increased local procoagulant activity in the airway and bronchoalveolar lavage fluid, while there was no change in plasma parameters [11]. The studies show that the procoagulant level is higher than normal in the early days of respiratory viral infections, and as the infections recover, the plasma level of procoagulant proteins returns to the baseline [15]. Accordingly, in our study, the procoagulant protein level should be high in the first blood samples taken and low in the second samples. However, although the second samples were taken 7–14 days later, there was no significant decrease in procoagulant parameters. As a result of the infection resolution over time, coagulation activity should decrease, not increase. Viruses cause platelet destruction in the acute phase of the infection. In the chronic phase of the infection, viruses have a negative effect on platelet production reducing platelet production. For this reason, viral infections are often accompanied by thrombocytopenia [12].The most important finding of our study is the increase in the platelet count in plasma. Despite viral infection in patients, erdosteine treatment has led to an increase in platelet count.

The decrease in the D-dimer level as a result of NAC treatment is a finding regarding the anticoagulant effect of NAC in this study. There are few *in vivo* and *in vitro* studies of the effects of NAC on coagulation in the literature. A group of researchers has found that coagulation factors and PT decreased significantly in six healthy male subjects who received 10 mg/kg NAC intravenously [2]. In their study, Knudsen et al. injected NAC intravenously at therapeutic doses to 10 healthy volunteers for 1–120 h [3]. They have found reduced levels of factors II, VII, IX, and XI, and therefore PT prolongation is related to the defect in the extrinsic pathway of the coagulation cascade. Jang et al. [16] have demonstrated a significant decrease in the activity of coagulation factors II, VII, IX, and X in the human plasma samples incubated with NAC. NAC has anticoagulant and platelet-inhibiting properties in the patients undergoing major surgery, which was associated with increased bleeding risk, blood loss, and blood product transfusion [4–5]. In a similar study, the need for reoperation increased significantly in the NAC group due to postoperative bleeding [17]. In the *in vitro* study of blood obtained from healthy volunteers, Pizon et al. [18] reported that increasing serum concentrations of NAC were associated with clinically significant prolongations in PT. Thorsen et al. [19] have demonstrated that NAC treatment prolongs PT through a direct effect on vitamin K-dependent clotting factors. The main reason why NAC reduces vitamin K-dependent clotting factors is probably through denitrosylating [20]. On the other hand, NAC can reversibly reduce disulfde bonds that are essential for the structure and function of clotting factors [21]. NAC can prevent thrombus formation in a dose-dependent manner without significantly affecting the bleeding time [22]. The study by Smyrniotis et al. [6] has also shown that NAC reduces platelet aggregation in rats. An increase in PT and INR and a decrease in platelet count occurred in the patient who was accidentally exposed to a high-dose (42 g) NAC infusion [23]. There may be chronic downregulation of the coagulation system through inhibition of von Willebrand factor function [24]. The thrombolytic effect appears to be mainly mediated by the cleavage of VWF, which cross-links platelets [25]. NAC was injected intravenously in the above-mentioned studies, but in the present study, NAC was given orally. The results obtained by intravenous administrations should not be directly adapted to patients receiving NAC orally because the oral bioavailability of NAC is much lower (approximately 9%) [26]. In the early hours (up to 38 h) after NAC administration, coagulation factor II+VII+X total activity decreased, and then the activity gradually returned to the baseline (by 120 h) [3]. In the present study, the treatment period was much longer than their studies (10±3 days vs 1–120 h).

Erdosteine affects both hemostasis and coagulation parameters in a dose-dependent manner [7]. Low-dose erdosteine (3 mg/kg) prolonged PT and INR in young rats and in tubes clotting takes place. They showed that hemostasis was impaired at the doses of 3 and 10 mg/kg/day, while no change occurred at higher doses (30 mg/kg/day). In another animal study, the researchers reported that factor II levels decreased in a dose-dependent manner in adult male rats treated with erdosteine [8]. In addition, INR was prolonged and platelet count increased in all groups, dose-independently. The findings of this study were similar to their results: factor II levels decreased and platelet count increased in male patients. It is highly likely that the effects of erdosteine on hemostasis are not related to gender. For example, in the study conducted on female rats, the researchers showed that erdosteine decreased the factor VIII level in a dose-dependent manner, and PT and APTT were prolonged [9]. As a result, the present data from aminal studies and this study suggest that the coagulation factor levels and platelet counts were impaired by erdosteine. There are almost no studies on humans in the literature investigating the effects of erdosteine on coagulation. Erdosteine is a prodrug that is rapidly absorbed after oral administration, converted to active metabolites via first-pass metabolism in the liver, and thus may affect the synthesis or degradation of clotting factors [27]. Therefore, its metabolites such as N-thioglycolyl-homocysteine (Met I), N-acetyl-homocysteine, and homocysteine formed as a result of first-pass elimination of erdosteine are important. Erdosteine is most commonly metabolized to the ring-opening compound Met I [28]. Erdosteine exerts both its pharmacological and clinical effects mainly through Met I, a homocysteine-based metabolite [29]. It is well known that high plasma homocysteine levels play an important role in the development of atherosclerotic and venous thromboembolic disease [30]. For example, PT is shortened in HIV patients with high homocysteine plasma levels, indicating an increased risk of thrombosis [31]. Therefore, homocysteine-based metabolites may be responsible for the effects of erdosteine on hemostasis and coagulation. In the experimental study, homocysteine increased the platelet count and fibrinogen level for a short period in rats [32]. Researchers also found a significant positive correlation of platelet count with serum homocysteine in hemodialysis patients with end-stage renal disease in two different studies [33]. Serum homocysteine has been significantly higher in hemodialysis patients than in controls, and homocysteine correlated directly with platelet count, PT, and INR, and inversely with APTT [34]. They suggested that high homocysteine induces the synthesis of platelets by an unknown mechanism and contributes to the incidence of thrombotic events among these patients. Increased homocysteine may also increase the aggregation of platelets by inducing adenosine diphosphate [35]. This means that the reason for hemostatic abnormalities caused by erdosteine is its metabolites containing homocysteine.

## Conclusion

In contrast to NAC, erdosteine may impair hemostasis, lead to a reduction in the level of coagulation factors, and an increase in platelet counts. We suggest that it should be considered when erdosteine is used together with anticoagulants, procoagulants, and other drugs affecting hemostasis. To prevent coagulation abnormalities in patients undergoing dental and medical operations, they should be questioned about erdosteine intake as well as the drugs that affect hemostasis. Secondly, erdosteine might be beneficial for patients presenting with thrombocytopenia because it increases the platelet counts and is well-tolerated.

## References

[1] Rogliani P. Matera M.G. Page C Puxeddu E. Cazzola M. Calzetta L. Efficacy and safety profile of mucolytic/antioxidant agents in chronic obstructive pulmonary disease: a comparative analysis across erdosteine, carbocysteine, and N-acetylcysteine. Respir Res 2019; 20(1): 104, 10.1186/s12931-019-1078-y 31133026 PMC6537173

[2] Jepsen S. Hansen A.B. The influence of N-acetylcysteine on the measurement of prothrombin time and activated partial thromboplastin time in healthy subjects. Scand J Clin Lab Invest 1994; 54(7): 543-547, 10.3109/00365519409088566 7863231

[3] Knudsen TT Thorsen S. Jensen S.A. Dalhoff K Schmidt L.E. Becker U. Bendtsen F. Effect of intravenous N-acetylcysteine infusion on haemostatic parameters in healthy subjects. Gut 2005; 54(4): 515-521, 10.1136/gut.2004.043505 15753537 PMC1774452

[4] Niemi T.T. Munsterhjelm E. Pöyhiä R. Hynninen M.S. Salmenperä M.T. The effect of N-acetylcysteine on blood coagulation and platelet function in patients undergoing open repair of abdominal aortic aneurysm. Blood Coagul Fibrinolysis 2006; 17(1): 29-34, 10.1097/01.mbc.0000195922.26950.89 16607076

[5] Wijeysundera D.N. Karkouti K. Rao V. Granton J.T. Chan C.T. Raban R. Carroll J. Poonawala H. Beattie W.S. N-acetylcysteine is associated with increased blood loss and blood product utilization during cardiac surgery. Crit Care Med 2009; 37(6): 1929-1934, 10.1097/CCM.0b013e31819ffed4 19384218

[6] Smyrniotis V. Arkadopoulos N. Kostopanagiotou G. Theodoropoulos T Theodoraki K. Farantos C. Kairi E. Paphiti A. Attenuation of ischemic injury by N-acetylcysteine preconditioning of the liver. Journal of Surgical Research 2005; 129(1): 31-37. 16140340 10.1016/j.jss.2005.07.028

[7] Arica V. Tutanc M. Ozturk O.H. Arica S. Basarslan F Erden E.S. Oktar S. Kaya H. Dual effects of erdosteine on hemostasis via its different metabolites in young rats. Hum Exp Toxicol 2011; 30(10): 1644-1648, 10.1177/0960327110396526 21247989

[8] Tutanc M. Arica V. Motor S. Basarslan F Erden E.S. Ozturk O.H. Zararsiz I. Aydin M. Effects of erdosteine on hemostasis: an experimental study. Hum Exp Toxicol 2012; 31(6): 574-578, 10.1177/0960327111426588 22045892

[9] Motor S. Alp H. Yuksel R. Erden E. Oktar S. Celik S. Cayirci G. Yilmaz E. The effects of erdosteine on coagulation in rats. Acta Medica Mediterranea 2014; 30: 801.

[10] Smeeth L Cook C Thomas S. Hall A.J. Hubbard R. Vallance P. Risk of deep vein thrombosis and pulmonary embolism after acute infection in a community setting. Lancet 2006; 367(9516): 1075-1079, 10.1016/S0140-6736(06)68474-2 16581406

[11] Majoor C.J. van de Pol M.A. Kamphuisen P.W. Meijers J.C. Molenkamp R. Wolthers K.C. van der Poll T Nieuwland R. Johnston S.L. Sterk P.J. Bel E.H. Lutter R. van der Sluijs K.F. Evaluation of coagulation activation after rhinovirus infection in patients with asthma and healthy control subjects: an observational study. Respir Res 2014; 15(1): 14, 10.1186/1465-9921-15-14 24502801 PMC3922343

[12] Assinger A. Platelets and infection — an emerging role of platelets in viral infection. Front Immunol 2014, 10.3389/fmmu.2014.00649 PMC4270245 25566260

[13] Bouwman J.J. Visseren F.L. Bosch M.C. Bouter K.P. Diepersloot R.J. Procoagulant and inflammatory response of virus-infected monocytes. Eur J Clin Invest 2002; 32(10): 759-766, 10.1046/j.1365-2362.2002.01041.x 12406025

[14] Goeijenbier M. van Wissen M. van de Weg C Jong E. Gerdes V.E. Meijers J.C. Brandjes D.P. van Gorp E.C. Review: viral infections and mechanisms of thrombosis and bleeding. J Med Virol 2012; 84(10): 1680–1696, 10.1002/jmv.23354 22930518 PMC7166625

[15] Keller T.T. van Wissen M. Mairuhu A.T. van Doornum G.J. Brandjes D.P. Acute respiratory tract infections in elderly patients increase systemic levels of hemostatic proteins. J Thromb Haemost 2007; 5(7): 1567–1569, 10.1111/j.1538-7836.2007.02580.x 17439634 PMC7166660

[16] Jang D.H. Weaver M.D. Pizon A.F. In vitro study of N-acetylcysteine on coagulation factors in plasma samples from healthy subjects. J Med Toxicol 2013; 9(1): 49-53, 10.1007/s13181-012-0242-2 22733602 PMC3576500

[17] Khalili F Khosravi M.B. Sahmeddini M.A. Eghbal M.H. Kazemi K Nikeghbalian S. Ghazanfar Tehran S. Khosravi B. The effect of perioperative N-acetylcysteine on the short and long term outcomes in pediatrics undergoing living-donor liver transplantation. Int J Organ Transplant Med 2021; 12(1): 12-20. PMC8717878 34987729

[18] Pizon A.F. Jang D.H. Wang H.E. The in vitro effect of N-acetylcysteine on prothrombin time in plasma samples from healthy subjects. Acad Emerg Med 2011; 18(4): 351–354, 10.1111/j.1553-2712.2011.01041.x 21496136

[19] Thorsen S. Teisner A. Jensen S.A. Philips M. Dalhoff K. Bendtsen F. Effect of N-acetylcysteine on the accuracy of the prothrombin time assay of plasma coagulation factor II+VII+X activity in subjects infused with the drug. Infuence of time and temperature. Scand J Clin Lab Invest 2009; 69(6): 643-650, 10.3109/00365510902943262 19530032

[20] Marley R. Patel R.P. Orie N. Ceaser E. Darley-Usmar V. Moore K. Formation of nanomolar concentrations of S-nitroso-albumin in human plasma by nitric oxide. Free Radic Biol Med 2001; 31(5): 688-696, 10.1016/s0891-5849(01)00627-x 11522454

[21] Kim K.Y. Rhim T. Choi I. Kim S.S. N-acetylcysteine induces cell cycle arrest in hepatic stellate cells through its reducing activity. J Biol Chem 2001; 276(44): 40591-40598, 10.1074/jbc.M100975200 11509553

[22] Bresette C.A. Ashworth K.J. Di Paola J. Ku D.N. N-acetyl cysteine prevents arterial thrombosis in a dose-dependent manner in vitro and in mice. Arterioscler Thromb Vasc Biol 2024; 44(2): e39-e53, 10.1161/ATVBAHA.123.319044 38126172

[23] Naraki K Mousavi S.H. Etemad L Rezazadeh-Shojaie S.M. Sadeghi T. Moshiri M. N-acetylcysteine overdose: a case report. International Journal of Medical Toxicology and Forensic Medicine 2021; 11(1): 32409, 10.32598/ijmtfm.v11i1.32409

[24] Bueche C.Z. Garz C. Kropf S. Bittner D. Li W Goertler M. Heinze H.J. Reymann K Braun H. Schreiber S. NAC changes the course of cerebral small vessel disease in SHRSP and reveals new insights for the meaning of stases — a randomized controlled study. Exp Transl Stroke Med 2013; 5: 5, 10.1186/2040-7378-5-5 23587288 PMC3661381

[25] Martinez de Lizarrondo S. Gakuba C. Herbig B.A. Repessé Y. Ali C. Denis C.V. Lenting P.J. Touzé E. Diamond S.L. Vivien D. Gauberti M. Potent thrombolytic effect of N-acetylcysteine on arterial thrombi. Circulation 2017; 136(7): 646-660, 10.1161/circulationaha.117.027290 28487393 PMC5560034

[26] Olsson B. Johansson M. Gabrielsson J. Bolme P. Pharmacokinetics and bioavailability of reduced and oxidized N-acetylcysteine. Eur J Clin Pharmacol 1988; 34(1): 77-82, 10.1007/BF01061422 3360052

[27] Dechant K.L. Noble S. Erdosteine. Drugs 1996; 52(6): 875-882, 10.2165/00003495-199652060-00009 8957158

[28] Cazzola M. Page C. Rogliani P. Calzetta L. Matera M.G. Multifaceted beneficial effects of erdosteine: more than a mucolytic agent. Drugs 2020; 80(17): 1799–1809, 10.1007/s40265-020-01412-x 33025535 PMC7647991

[29] Cattò C Villa F. Cappitelli F. Understanding the role of the antioxidant drug erdosteine and its active metabolite on staphylococcus aureus methicillin resistant bioflm formation. Antioxidants (Basel) 2021; 10(12): 1922, 10.3390/antiox10121922 34943025 PMC8698571

[30] Ray J.G. Meta-analysis of hyperhomocysteinemia as a risk factor for venous thromboembolic disease. Arch Intern Med 1998; 158(19): 2101-2106, 10.1001/archinte.158.19.2101 9801176

[31] Roca B. Roca M. Girones G. Increased homocysteine plasma level is associated with shortened prothrombin time in HIV-infected patients. HIV Clin Trials 2016; 17(5): 218-223, 10.1080/15284336.2016.1220712 27561455

[32] da Cunha A.A. Scherer E. da Cunha M.J. Schmitz F. Machado F.R. Lima D.D. Delwing D. Wyse AT. Acute hyperhomocysteinemia alters the coagulation system and oxidative status in the blood of rats. Mol Cell Biochem 2012; 360(1-2): 205-214, 10.1007/s11010-011-1058-0 21948259

[33] Nasri H. Impact of serum homocysteine on platelet count in stable hemodialysis patients. The Journal of Applied Research 2006; 6(1): 14-18.

[34] Yassin M. Lubbad A.M. Abu Taha A. Saadallah N. Homocysteine and hematological indices in hemodialysis patients. Ibnosina Journal of Medicine and Biomedical Sciences 2014; 06(04): 173-179, 10.4103/1947-489x.210380

[35] Zanin R.F. Campesato L.F. Braganhol E. Schetinger M.R. Wyse AT. Battastini A.M. Homocysteine decreases extracellular nucleotide hydrolysis in rat platelets. Thromb Res 2010; 125(3): e87-92, 10.1016/j.thromres.2009.09.020 19850326

